# Flash Thermal Racemization of Chiral Amine in Continuous Flow: An Exploration of Reaction Space Using DoE and Multivariate Transient Flow

**DOI:** 10.1021/acs.oprd.4c00508

**Published:** 2025-02-11

**Authors:** Matthew J. Takle, Linden Schrecker, Benjamin J. Deadman, Joachim Dickhaut, Andy Wieja, Klaus Hellgardt, King Kuok Mimi Hii

**Affiliations:** † Department of Chemistry, Molecular Sciences Research Hub, Imperial College London, 82, Wood Lane, London W12 0BZ, U.K.; ‡ Centre for Rapid Online Analysis of Reactions, Molecular Sciences Research Hub, Imperial College London, 82, Wood Lane, London W12 0BZ, U.K.; § BASF SE, Ludwigshafen 67056, Germany; ∥ Department of Chemical Engineering, Imperial College London, Exhibition Road, South Kensington, London SW7 2AZ, U.K.

**Keywords:** flash thermal racemization, flow chemistry, DoE, transient flow, data-driven, reaction mapping

## Abstract

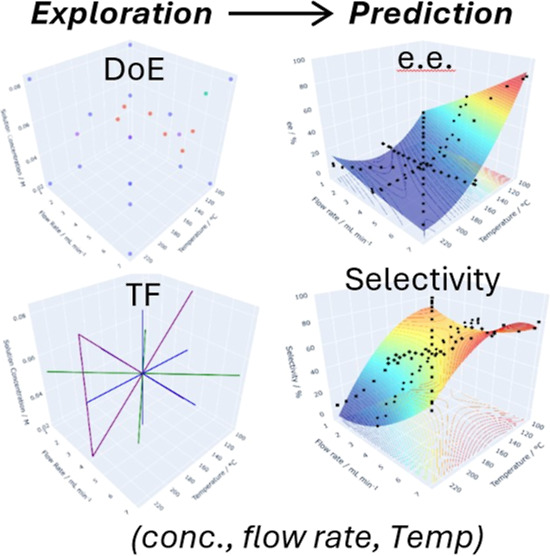

The robustness of the flash thermal racemization of optically active 1-phenylethylamine over Pd/γ-Al_2_O_3_ was studied by applying split-plot design-of-experiments (DoE), where the effects of temperature, flow rate, and concentration (3-factors) on the e.e. and selectivity (2-responses) were examined and quantified. The same effects were also interrogated using multivariate ramps in transient flow to produce response surfaces for the reaction space. The same set of optimal conditions for the process was identified by both approaches, and the same relationships between the variables were observed: while the extent of racemization (e.e.) can be directly correlated to temperature, a more complex relationship between temperature and flow rate on the selectivity was uncovered.

## 1 Introduction

A chemical reaction involves several closely associated discrete and continuous variables that work synergistically to affect the reaction outcome, e.g., reactant stoichiometry, catalyst, pH, additives, solvent, temperature, and pressure. The “robustness” of a chemical reaction is the ability to tolerate changes in these variables without deleterious effects. Understanding how the variables affect the reaction outcome is not only important in understanding the mechanism but also critical in designing an appropriate process that can guarantee a consistent product quality (quality by design, QbD).^1,2^

Most scientists and engineers were trained to perform one-factor-at-a-time (OFAT) experiments, where the effect of one factor (or variable) is studied while keeping the other factors at fixed values. OFAT can be effective for optimizing the yield of a simple reaction with few variables and is generally favored by academic researchers as the experiments are simple to perform and the results can be easily interpreted, qualitatively, by chemical intuition.

In the past few decades, however, OFAT has been largely eschewed by industrial R&D laboratories, in favor of statistical methods for reaction optimization, specifically multivariate and multiobjective analyses.^3^ Among various approaches, design-of-experiments (DoE) is extensively adopted for implementing QbD,^4,5^ although Bayesian algorithms are also becoming increasingly popular.^6−8^ Both DoE and Bayesian optimization methods aim to increase the efficiency of experimentation, particularly when there are multiple interacting variables. Meanwhile, advances in automation, high-throughput experimentation, and analytical tools^9^ have greatly simplified the acquisition of experimental data, which, in turn, improves the efficiency and accuracy of statistical models. In this regard, we developed new transient flow (TF) methods to collect multiparameter data of homogeneous reactions. Initially, the TF method involved varying the flow rate of reactants in a controlled step change or linear ramp to gather residence time data.^10^ In recent years, this concept has been extended to temperature or concentration gradients that can afford additional kinetic data to support the development of kinetic models^11^ and delineate substituent effects (Hammett parameters).^12^

**Scheme 1:**
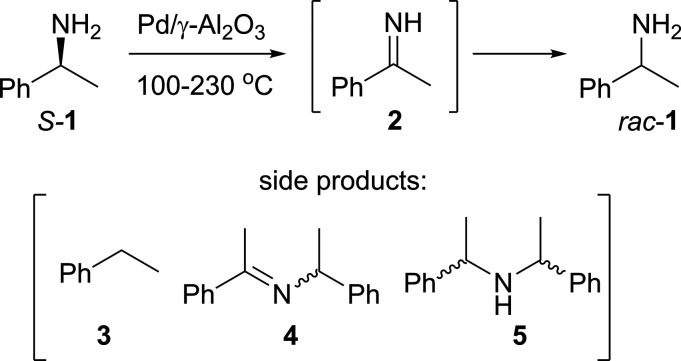
Racemization of *S*-**1** over Pd/γ-Al_2_O_3_ via Imine Intermediate **2** and Possible Side Products (**3**–**5**)

Recently, we reported a more sustainable and scalable continuous flow strategy for chemoenzymatic dynamic kinetic resolution (CE-DKR) of chiral amines.^13^ Key to this methodology is the development of an additive-free method of racemizing a chiral amine, by exposing it over a heterogeneous catalyst (Pd/γ-Al_2_O_3_) at high temperatures in just a few seconds: using residence time control to effect the racemization without competitive side product formation, a process we termed “flash thermal racemization” (FTR). The racemization involves a series of reversible H-transfers between the primary amine and the transition metal via an imine intermediate **2**, which can produce unwanted side-products **3**–**5** (Scheme 1).

The aim of this work is to study and delineate the effects of three reaction variables (flow rate, temperature, and concentration) on the e.e. and selectivity of the FTR of a chiral amine over a packed bed of Pd catalyst (Scheme 1) using DoE and TF methods. The applicability of the two different approaches in assessing the overall robustness of the process was evaluated. Comparisons between the experimental approaches (steady-state vs TF) as well as the quantity and quality of the outputs will be made to highlight the value of each approach for different objectives. TF methods had been widely adopted in heterogeneous catalysis for gas-phase reactions over solid catalysts.^14^ Thus far, however, there is only one gas–liquid example where the hydrogenation of an aryl ketone over catalytic static mixers was studied by ramping flow rate.^15^ Herein, bivariate TF ramping (flow rate–temperature and flow rate–concentration) will be demonstrated for the first time on a heterogeneous catalytic system involving a solid–liquid interface.

## 2 Results and Discussion

In the present work, the robustness of the FTR methodology will be investigated by subjecting an optically pure 1-phenethylamine (*S*)-**1** to high temperatures over a Pd/Al_2_O_3_ catalyst at different flow rates, corresponding to residence times, *t*_Res_, between 9 and 63 s. The effectiveness of the racemization process can be measured by determining the enantiomeric excess (e.e.) of **1** (eq 1), whereas the selectivity for the primary amine is calculated by determining the amount of **1** retained (eq 2). In a FTR reaction, the overall aim is to minimize the e.e. (→0%) while retaining high selectivity (→100%) for the primary amine.
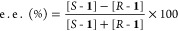
1
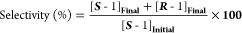
2

## 3 DoE Campaign

**Figure 1:**
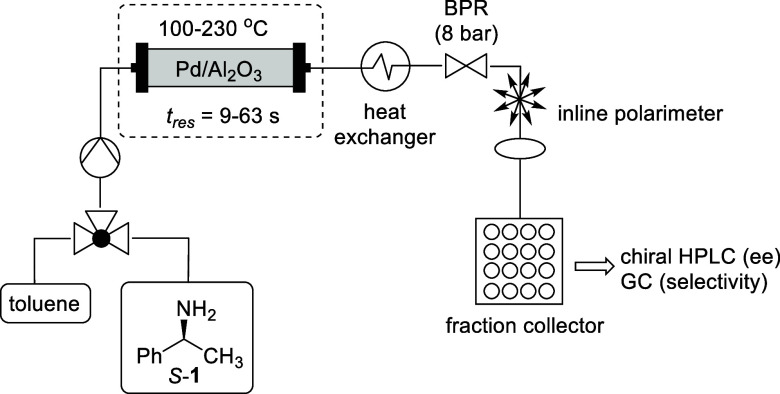
Flow reactor configuration utilized for the DoE work.

DoE and flow chemistry work well together as complementary tools as the short residence times allow the data to be collected in a timely manner.^16,17^ The DoE experiments were performed using the reactor configuration described in our original work (Figure 1),^13^ where each independent variable was adjusted to a preset value and maintained throughout the experiment. When the system reached steady state, indicated by a stable reading on an in-line polarimeter, reaction aliquots were collected using a fraction collector and subsequently analyzed by GC (selectivity) and chiral HPLC (e.e.).

The design contains three inputs (“factors”): reaction temperature (100–230 °C), flow rate (1–7 mL min^–1^), and initial amine concentration (20.6–82.5 mM) and two outputs (“responses”): e.e. and selectivity. In this work, JMP’s DoE software was used to construct an I-optimal split-plot response surface design, composed of 18 experiments (Table S1, entries 1–18, Supporting Information). This protocol was used to screen the defined reaction space for main effects, as well as quadratic and second order interactions between the input variables. From here, the influence of the reaction parameters on the reaction outcomes can be analyzed using analysis of variance (ANOVA) and linear regression to identify the parameters that are statistically significant.

Due to the design of the reactor system, where only one pump was used to deliver either toluene or the amine solution (via a 3-way valve, Figure 1), changing the solution concentration, [*S*-**1**], will involve replacement of the feedstock solution and flushing the reactor. Indeed, it is best practice to replace the feedstock between each experiment (even if it is equivalent) to ensure they are truly independent data points. While such practices are statistically preferable, they are inconvenient and slow to conduct due to the down time between experiments. The DOE solution to this inflexibility is to implement a split-plot design, whereby the experiment set was divided into whole plots, with [*S*-**1**] being kept constant within each plot. Using the split-plot I-optimal design removes the need to fully randomize experiment order while still testing the effect of [*S*-**1**] and allows the variation between the whole plots to be evaluated, albeit with a reduced statistical power compared to other variables. To monitor for catalyst deactivation, the first reaction within each plot was replicated at the end of the plot to check for reproducibility. While this design was our preferred option for this study, it should be noted that other designs are possible and the challenge of DoE is to customize the design to match the experimental needs, in the presence of uncertainty.

The initial results obtained from the split-plot design revealed that the statistically most significant effect (*P*-value < 0.05) affecting e.e. is temperature (Table 1), while the selectivity is influenced by temperature and flow rate (Table 2). The amine concentration, [*S*-**1**], does not appear to be a significant effect for either response, but it should be noted that the split-plot design was known to have reduced power for testing this effect,^18^ and consequently only a larger effect would be expected to pass the significance test. Running additional whole plots would increase the power to detect the hypothesized effect.

**Table 1:** Split-Plot Design Screen Effects Summary for e.e.

entry	reaction parameter	*P*-value
1	temp	<0.00001
2	temp × temp	<0.00001

**Table 2:** Split-Plot Design Screen Effects Summary for Selectivity

entry	reaction parameter	*P*-value
1	temp	0.00001
2	flow rate	0.00241
3	temp × flow rate	0.01910

**Figure 2:**
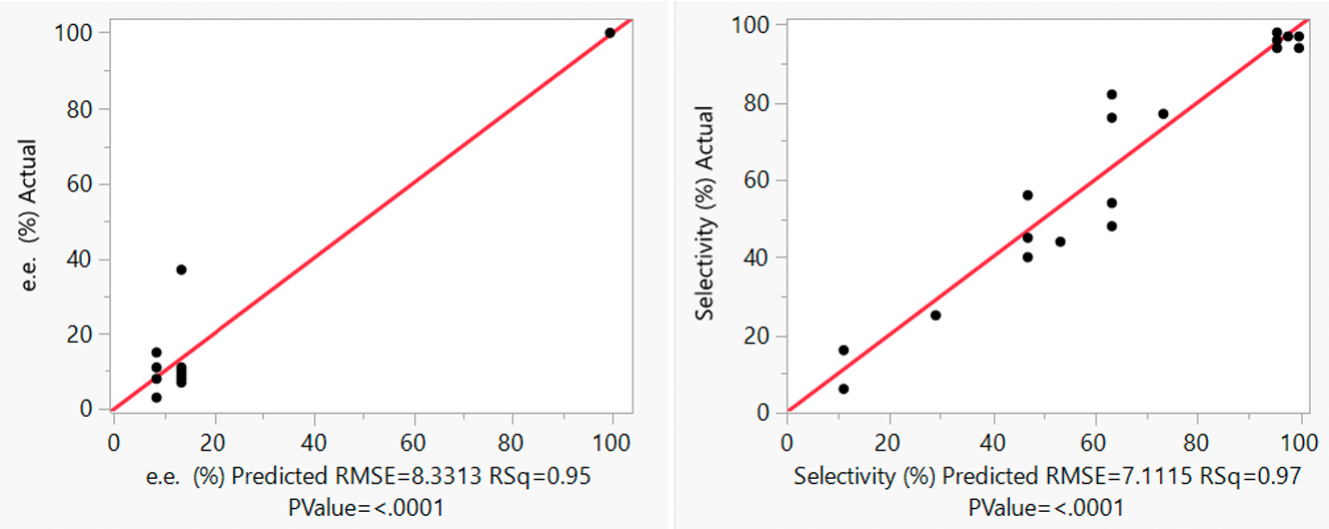
Actual by predicted plots for the split-plot design for the FTR of *S*-**1** with respect to e.e. (left) and selectivity (right).

**Figure 3:**
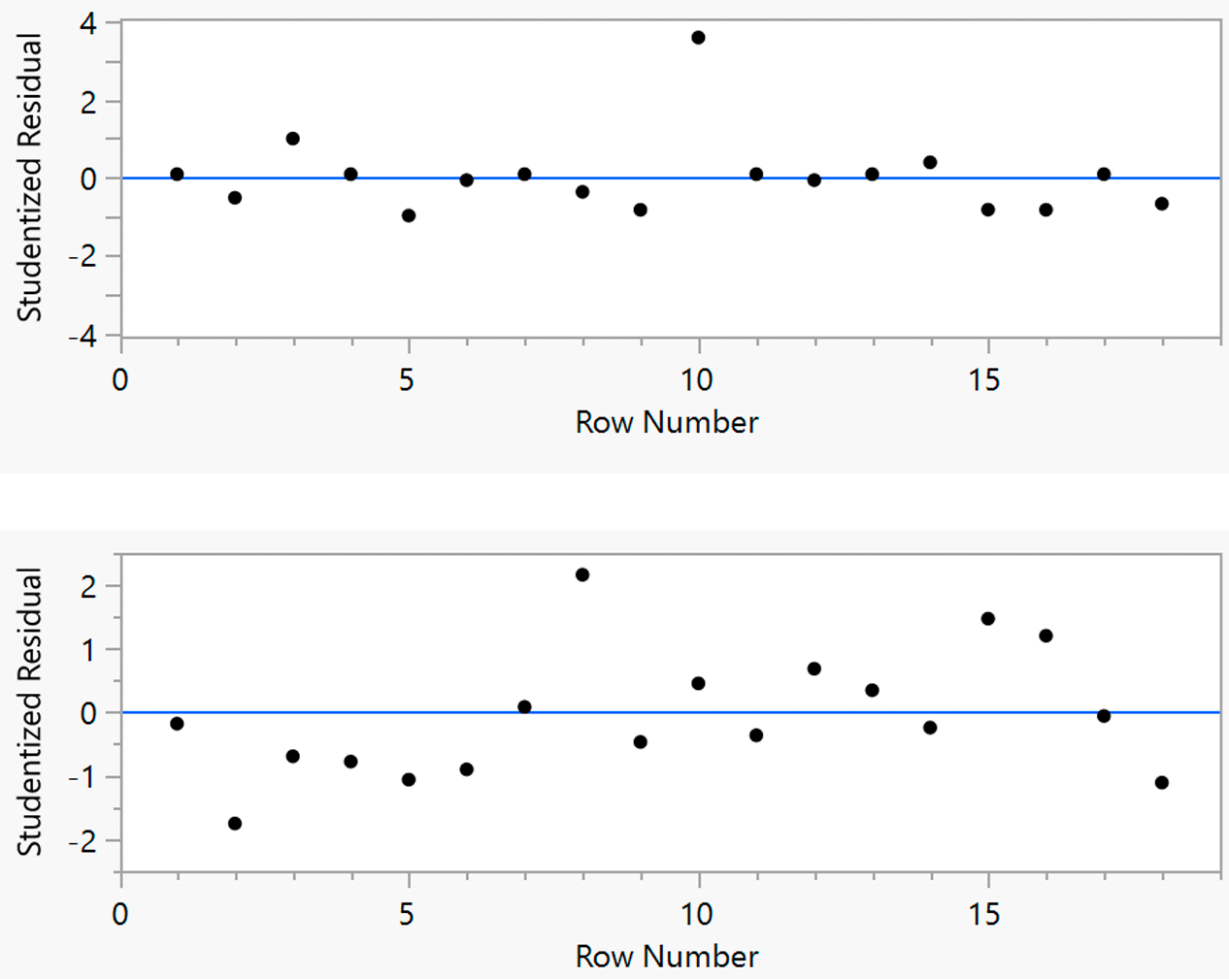
Split-plot design results for studentized residuals for the FTR of *S*-**1** with respect to e.e. (top) and selectivity (bottom).

The performance of the regression model is assessed by actual vs predicted plots (Figure 2), which displayed good *R*^2^ values of 0.96 (±0.01). However, the RMSE values (8.3 and 7.1) are higher than is typically desirable, indicating a high degree of unexplained noise in the experimental data. Furthermore, the ee values can be seen to cluster around 0–20% and 100%, which gives a weaker description of the reaction space across a range of e.e. values. Finally, the studentized residuals are largely randomly distributed within ±3 range around the center point 0 (Figure 3). This demonstrates the absence of systematic errors.

**Figure 4:**
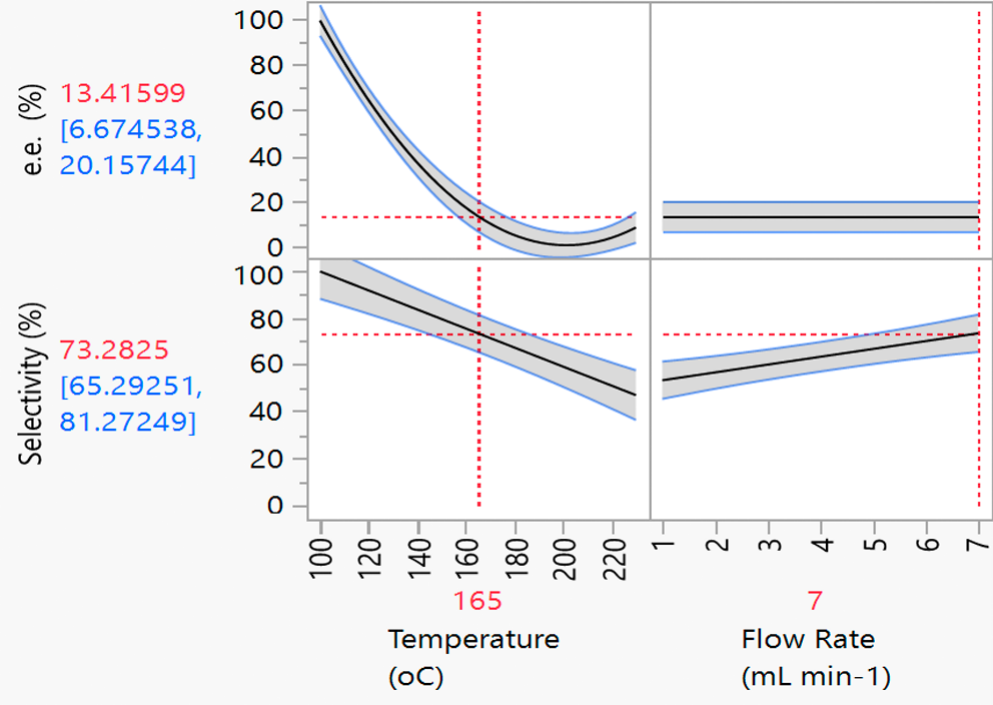
Split-plot design Prediction Profiler for the FTR of *S*-**1**.

The best practice in DOE is to use sequential experimental designs to add complexity to the model. In the current study, the first round of DOE gave a coarse model of the reaction. To increase the predictive power of the model with respect to e.e., a greater number of data points in the 20–90% e.e. range were needed. To achieve this, the profile predictor (Figure 4) suggested that temperatures between 100 and 165 °C and flow rates of 1–7 mL min^–1^ should be investigated. As the e.e. does not appear to be strongly dependent on [*S*-**1**], a midpoint concentration (51.6 mM) was chosen for this part of the DoE work.

Using the augmented design, a further 6 key data points were added to the model using a space filling algorithm (Table S1, entries 19–24, Supporting Information). These extra data allowed for previously unobserved relationships between reaction inputs and outputs to be observed (Tables 3 and 4). Once again, temperature was found to be the predominant effect on both e.e. and selectivity (Table 3, entry 1 and Table 4, entry 1), while flow rate appears to exert a more significant effect on the selectivity than it does on the e.e. (Table 3, entry 3 vs Table 4, entry 2).

**Table 3:** Augmented Design Screen Effects Summary for e.e.

entry	reaction parameter	*P*-value
1	temp. (*T*)	0.00001
2	temp × temp (*T* × *T*)	0.00001
3	flow rate (*Q*)	0.04913
4	temp. × flow rate (*T* × *Q*)	0.08189

**Table 4:** Augmented Design Screen Effects Summary for Selectivity

entry	reaction parameter	*P*-value
1	temp (T)	0.00001
2	flow rate (*Q*)	0.00022
3	temp × flow rate (*T* × *Q*)	0.05205

**Figure 5:**
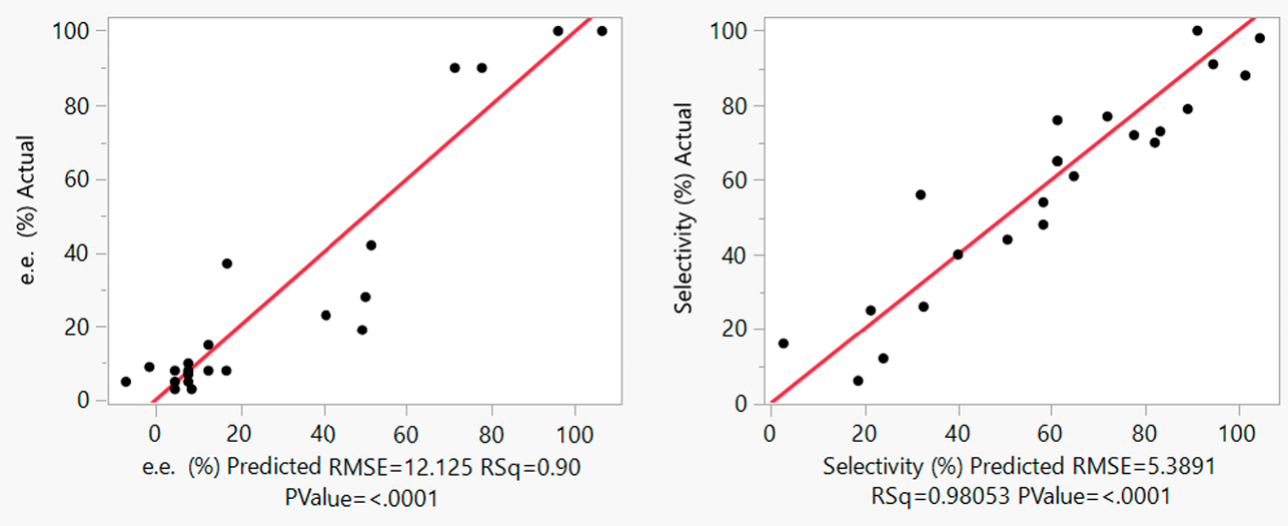
Actual vs predicted plots for the augmented design for the FTR of *S*-**1** with respect to e.e. (left) and selectivity (right).

**Figure 6:**
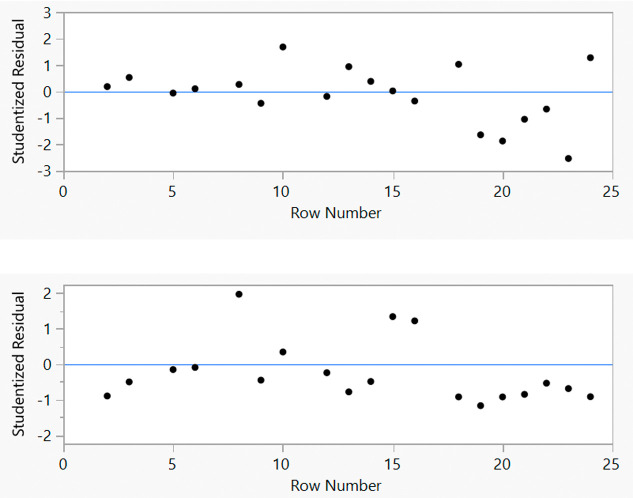
Augmented design results for studentized residuals for the FTR of *S*-**1** with respect to e.e. (top) and selectivity (bottom).

Further inspection of the actual vs predicted plots shows that the additional data were within the desired range of 20–90% e.e (Figure 5), giving better predictability across the reaction space: all the studentized residuals now falling within the acceptable ±3 range (Figure 6).

Finally, the accuracy of the DoE model was evaluated by a set of validation experiments: three sets of reaction conditions were generated randomly within the reaction space, and the predicted e.e. and selectivity values were compared to experimental results. Pleasingly, all the predictions were accurate within the expected error (Table 5, entries 1–3). Subsequently, the DoE model was used to identify the optimal conditions within the design space (Table 5, entry 4): by exposing an optically pure solution of (*S*)-**1** in toluene (82.5 mM) to a packed bed containing 10 mg of Pd/γ-Al_2_O_3_ at 140 °C for 9 s@7 mL/min, the e.e. of the chiral amine decreases from 100 to 42% upon a single-pass while retaining 95% selectivity for the primary amine.

**Table 5:** Augmented Design Model Prediction of Reaction Outcomes of the FTR of *S*-**1**^a^

entry	*T* (°C)	F (mL min^–1^)	[*S*-**1**] (mM)	pred. e.e. (%)^b^	obs. e.e. (%)^c^	pred. sel. (%)^b^	obs. sel. (%)^d^
1	165	4	51.6	8 (±11)	5	63 (±8)	65
2	125	5.5	51.6	70 (±20)	90	88 (±9)	99
3	200	2	51.6	0 (±12)	<5	36 (±8)	26
4^e^	140	7	82.5	51 (±14)	42	95 (±13)	91

a All experiments were performed with a solution of optically pure (*S*)-**1** in anhydrous toluene.

b Values in parentheses give the error range predicted by the model.

c Determined by chiral HPLC.

d Determined by GC.

e Optimal conditions predicted by the model.

**Figure 7:**
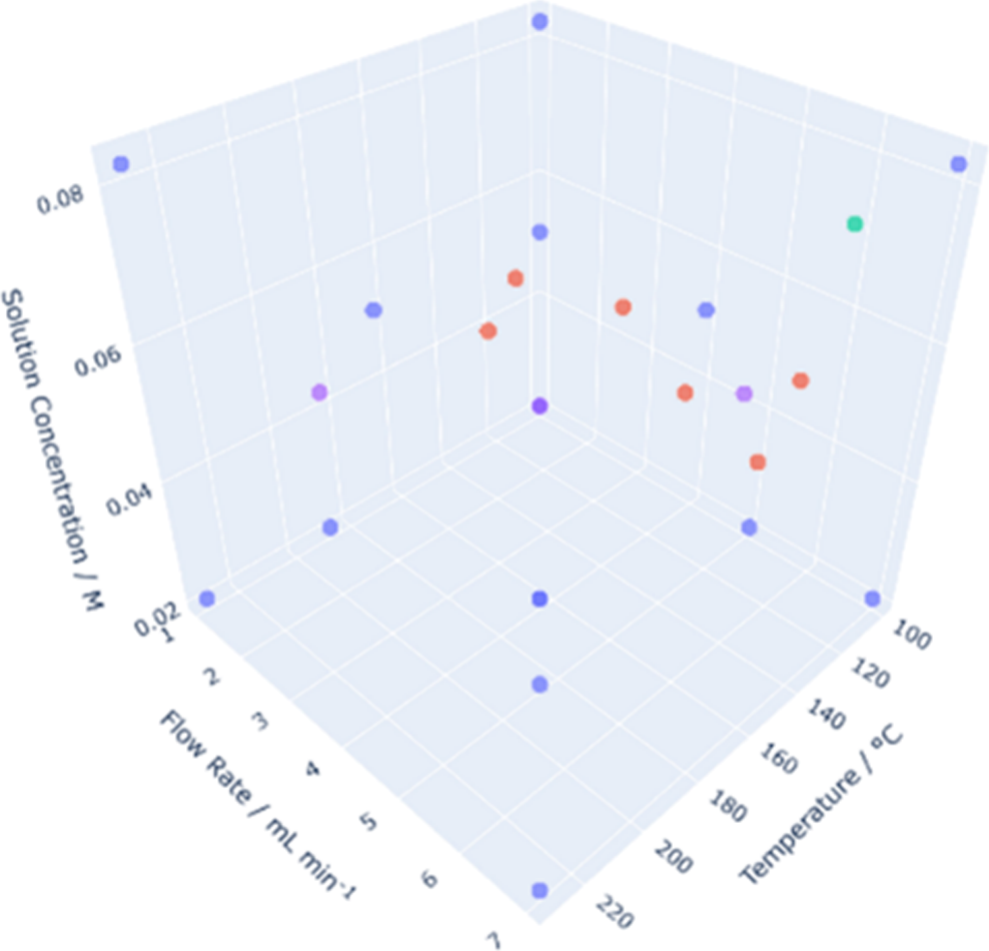
Distribution of sample points collected for the DoE experiments in the chosen design space; separated into initial (split-plot, blue), augmented (red), validation (purple) experiments, and the proposed optimum (green).

The distribution of sampling points collected for the DoE study is shown in Figure 7. The initial design occupies the edges and the midpoint of the design space (blue circles). The augmented design added 6 data points (red circles), closely clustered around the temperature-flow region where the widest variance in ee was found. The final three validation points (purple circles) were selected from different flow rates and temperatures, keeping the substrate at 51.6 mM, and the final optimal point (green circle) was found at the maximum concentration and flow rate of the design space but at a moderate temperature (140 °C).

## 4 TF Campaign

**Figure 8:**
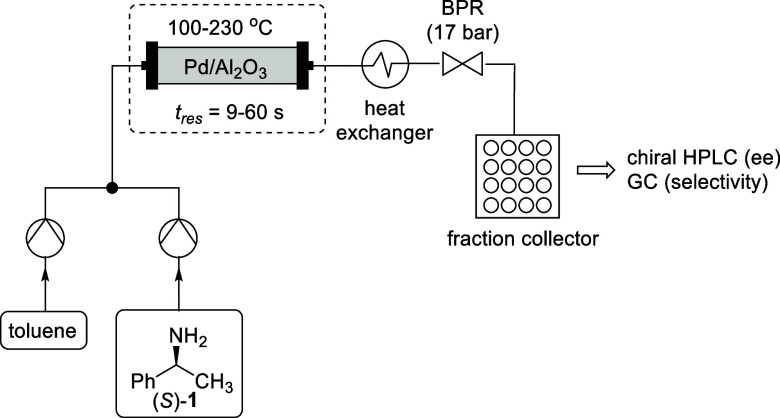
Modified FTR reactor employed for TF experiments.

Modifications to the reactor system were made to explore the reaction space by TF methods (Figure 8, details provided in Supporting Information). Toluene and a solution of *S*-**1** were each dispensed with a programmable piston pump into a T-piece to allow different concentrations and gradients to be generated in-line. To execute temperature ramps, the pack-bed reactor was heated by using a programmable GC oven. Lastly, the in-line polarimeter was removed as the monitoring of steady state is no longer required.

The three continuous variables described above in the DoE studies were reexamined using TF methods. Initially, the studies were conducted using monovariate ramps, i.e., by setting two of the variables to their midpoint values and varying the remaining variable across its entire range of the design space (Sections S2.1–2.4, Supporting Information).

Each ramp includes the midpoint of the design space, which serves as a useful benchmark between the experiments. The accuracy of the results was further verified by applying monovariate temperature and amine concentration gradients in both directions (“ramp up” and “ramp down”) over the same range. For variable flow rate experiments, ramping of flow rate was only applied in one direction (downward, “reverse push out’) as this was previously found to afford more accurate results. The absence of hysteresis between the data points collected in either direction confirmed the validity of the ramping method as well as catalyst stability over the experiment.

**Figure 9:**
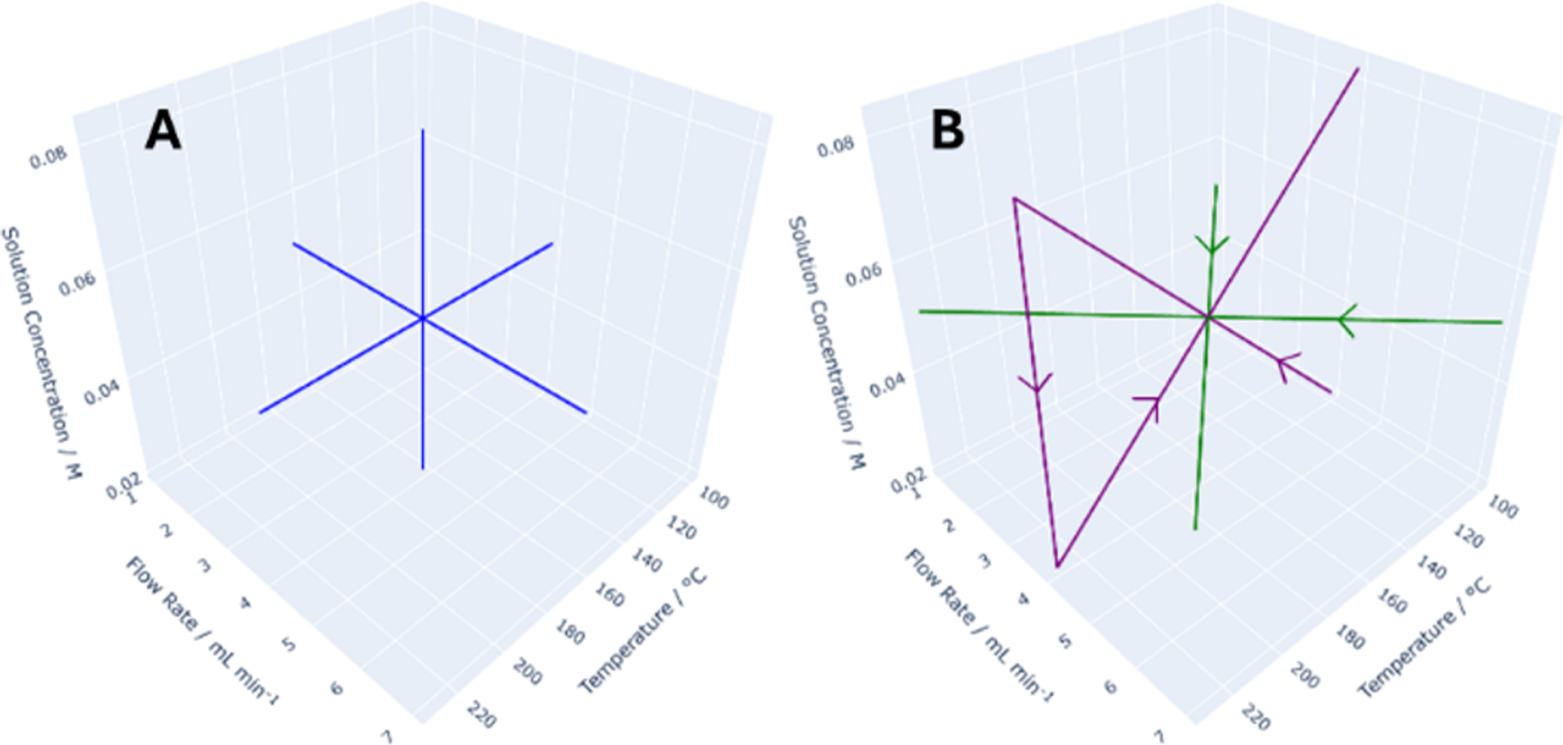
Mono-(A) and bi-(B)variate TF ramps.

The monovariate ramps allow us to perform essentially a series of OFAT experiments, providing orthogonal data points within the design space in a continuous manner (Figure 9A). To test for interactions between the variables, bivariate ramps were implemented in the same design space, enabling us to map the reaction spaces that are diagonal to the data generated by the monovariate ramps (sections S2.5 and S2.6, Supporting Information), specifically, temperature with flow rate (green lines, Figure 9B) or with amine concentration (purple lines, Figure 9B). Once again, the midpoint of the design space where the two bivariate ramps intersect serves as a benchmark to verify the absence of catalyst deactivation between the experiments.

**Figure 10:**
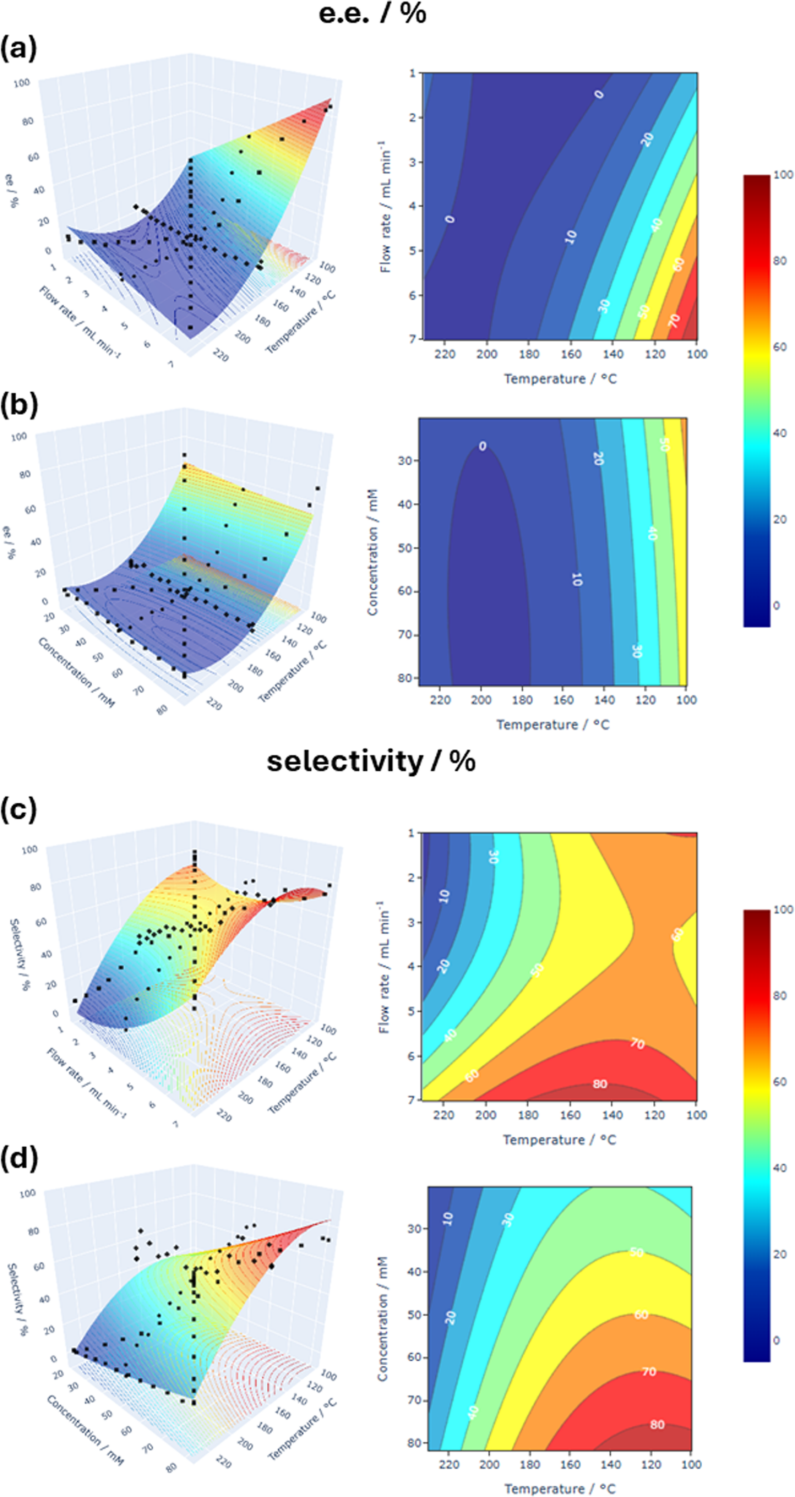
TF data collected across mono- and bivariate ramps. Response surfaces (left) and contour plots (right) of the e.e. (a,b) and selectivity (c,d) models (second order) fitted to the TF data are also visualized on these plots.

The TF data captured using mono- and bivariate ramps were fitted to a multivariate (second order) polynomial regressor model to describe the ee and selectivity (Table S6, Supporting Information). The models were subsequently used to produce response surface area and contour plots (Figure 10; a link to interactive versions of these plots is provided in the Supporting Information). For the racemization process, the plots comprise steep slopes (closely spaced contour lines) along the temperature axis, commensurate with a large response of e.e. to varying reaction temperature, particularly between 100 and ca. 140 °C (Figure 10a,b). Meanwhile, a positive correlation between flow rate and temperature was observed (Figure 10a); i.e., racemization is promoted by an increase in temperature and decrease in flow rate. In comparison, no such gradient was observed in the temperature-[*S*-**1**] plot (Figure 10b); i.e., the racemization is largely independent of amine concentration, confirming the earlier results obtained by DoE.

Conversely, while the selectivity of the FTR is also strongly temperature dependent, the relationships between flow rate-temperature (Figure 10c) and [*S*-**1**]-temperature (Figure 10d) are more complex as all three reaction variables affect the formation of side products, resulting in observed saddle (Figure 10c, orange region) and peak (Figure 10d, dark red region) features in the contour plots. In this case, high selectivity of >80% for the primary amine is limited to a small region in the reaction space between 120 and 150 °C, where [*S*-**1**] and flow rate are high.

Comparing the response surface area plots for e.e. and selectivity, it is evident that it is not possible to achieve both 0% e.e. and 100% selectivity within this reaction space (at least not within a single pass). As the overall CE-DKR process will operate in a recycle-batch mode, with multiple passes of the chiral amine through the FTR reactor to achieve complete conversion to the amide product, it is more important to prioritize selectivity over e.e. Indeed, the optimized conditions (140 °C, 82.5 mM of *S*-**1,** and flow rate of 7 mL min^–1^), identified earlier by the DoE campaign, do appear to offer the “sweet spot”, affording an appreciable decrease in e.e. with minimum side product formation. The ability to retain high selectivity for the primary amine at high flow rates and amine concentrations is also very encouraging for the potential scalability of the process. In this context, the stability of the Pd/Al_2_O_3_ catalyst against deactivation during these studies is particularly notable.

**Figure 11:**
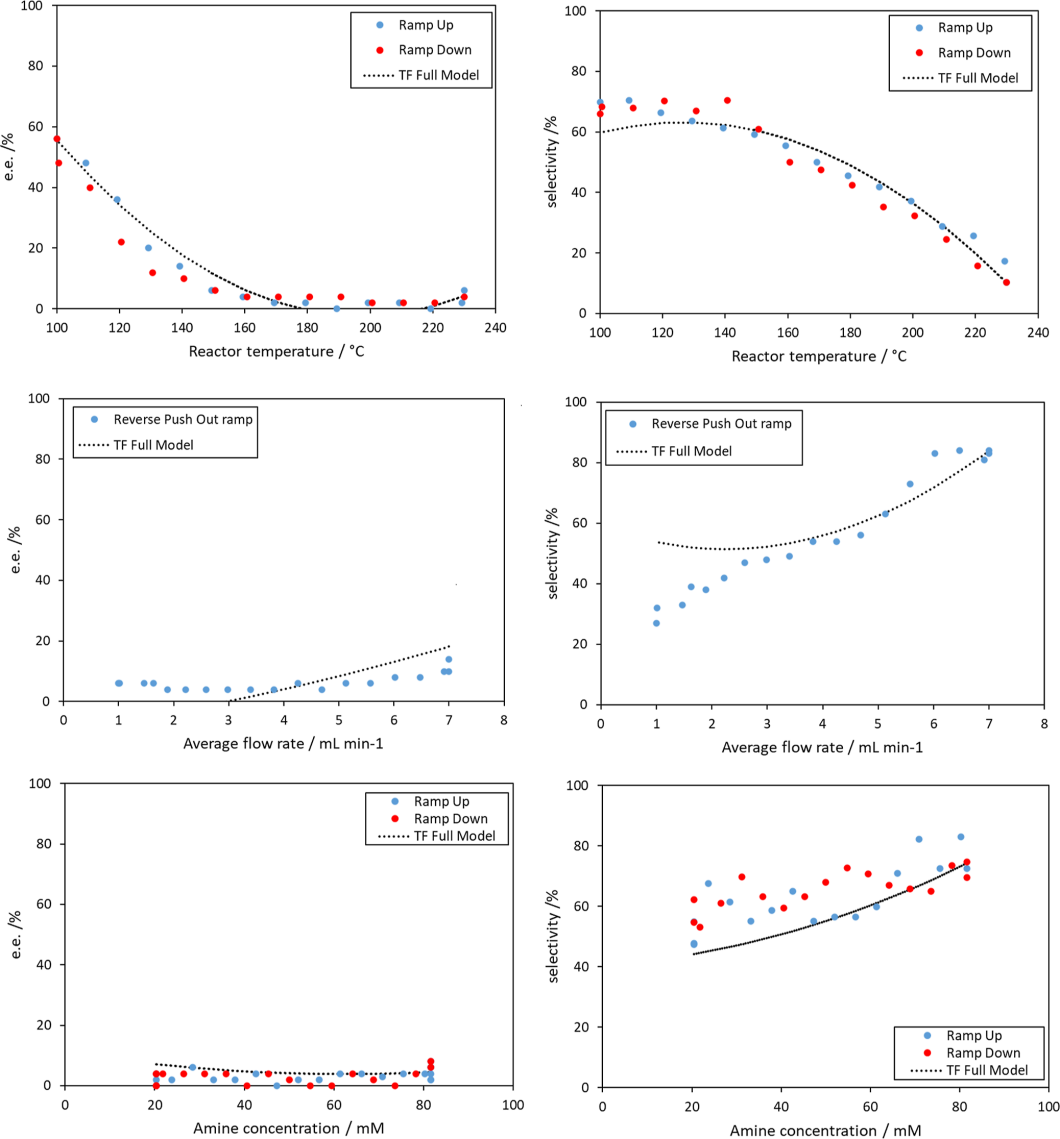
Comparison of the second-order polynomial model vs monovariate TF results.

The accuracy of the model generated by mono- and bivariate TF flow is assessed by comparing the model prediction on interactive surface plots (Figure 10) and predictions of the monovariate ramps (Figure 11). The second-order multivariate polynomial regression models produce *R*^2^ values of 0.92 and 0.85 and RMSE values of 5.3 and 9.3, respectively, for e.e. and selectivity over the four-dimensional model space (Table S7, Supporting Information). Attempts to fit the data to a higher third-order polynomial (Figure S8 and Table S8, Supporting Information) resulted in an expected increase in *R*^2^ values of 0.96 and 0.88, and RMSE values of 3.8 and 8.3, for e.e. and selectivity, respectively; i.e., a change from a quadratic to a cubic polynomial resulted only in marginal improvements to the quality of the fit. Considering that these models are fitted empirically and do not encode any chemical logic, the simpler model is considered to be adequate in this case in order to avoid overfitting. The deficiencies of this “black box” method can be observed in the predictions of monovariate ramps (Figure 11), especially for the flow rate ramp predictions, for example, demonstrating unrealistic e.e. values (<0%). Fitting of these data to a multiphysics model will be the subject of future work.

## 5 Comparison of DoE and TF Methods

The data obtained from the monovariate ramp TF experiments were compared with the results predicted by the DoE model. Overall, the correlations revealed previously by the DoE study are corroborated by the TF experiments; i.e., temperature exerts the largest effect on both selectivity and e.e., flow rate has a bigger effect on selectivity than e.e., and amine concentration ([*S*-**1**]) has a negligible effect on both.

Both models show that increasing temperature generally lowers the e.e. and selectivity. The ability to apply bivariate ramps allows better interrogation of complex secondary effects (e.g., as shown in Figure 10c) and a better definition of the reaction space. In this case, the split-plot design of the DoE study did not allow the effect of amine concentration [*S*-**1**] to be interrogated thoroughly, but this was confirmed more convincingly by the TF study. Both DoE and TF methods lead to the identification of optimal reaction conditions for the FTR process within the design space.

The practical aspects of DoE and TF experimentations were also compared, including the quantity of chemicals (chiral amine and solvent) needed to perform the experimental campaigns as well as the total amount of operating time required to run the experiments and to obtain the data (Table 6). Compared to DoE, the application of multiple ramps under dynamic flow required by the TF method required ca. 22% more amine and more than twice the amount of solvent. Hence, the TF method demands more material, which may be disadvantageous if the process involves expensive or limited feedstocks.^11^ In terms of the total amount of time required to perform the experiments, this is more nuanced: Using the flow systems described in Figures 1 and 8, a total of 12 h (over 1.5 days) was needed to perform the DoE experiments and acquire the data points, while the TF experiments only required 6 h of run time following setup. The time required to perform the DoE experiments can be shortened by the introduction of automation; in this case, the most time-consuming step will be the time required for the system to attain a steady-state conversion. Conversely, while the TF system is inherently more automated, the number of data points was much greater (albeit with the limitation that the gradient data points are not truly independent). In this study, the analytical samples were prepared by using an automated liquid handler. More time can be saved if in-line analytics can be introduced; i.e., the speed of the analytical method will determine the overall operation time for TF experiments.

**Table 6:** Comparison of Operation Parameters for DoE and TF Campaigns

parameter	DoE	TF
operating time	12 h	6 h
amine consumed	5.71 g	6.98 g
solvent usage	548 mL	1130 mL

## 6 Conclusions

The FTR of 1-phenylethylamine over Pd/Al_2_O_3_ was studied, where the effects of three reaction parameters (temperature, flow rate, and concentration) on the e.e. and selectivity of the process were quantified using DoE and TF approaches. Both approaches yielded models that predicted the same optimal conditions within the design space, with temperature as the dominant factor for the racemization, while high selectivity for the primary amine is achievable at high flow rates and concentrations.

Using a split-plot design, the DoE method can elucidate the statistically significant parameters and predict the optimal conditions with only 27 experimental data points. While expert knowledge is generally not required, chemical intuition was required to select factor ranges that were wide enough to detect the experimental effect, without being so wide that the results are a binary choice between 0% or 100% selectivity or e.e. In this case, further experiments (augmented design) were needed to enhance the e.e. prediction.

In contrast, TF experiments do not require as much expert chemical knowledge or mechanistic assumptions to select factor ranges since the transient tests a continuum of factor settings. Using bivariate ramps, a large amount of diverse empirical data could be collected relatively quickly within the reaction space with greater efficiency. This allows an objective model to be constructed without any human bias. However, the large number of data points can potentially hide the limited independence of the measurements within gradients. Careful examination of the data is required to avoid experimental errors manifesting as a systematic bias in the model.

The use of TF for reaction exploration is also relatively new, for which there is currently no bespoke software, requiring users to be conversant with programming.^19^ The TF setup will also require a higher degree of automated control than that offered by current commercial flow systems, which can create a higher barrier to entry.

In summary, DoE can be an efficient method for obtaining simple response surface models without specific equipment requirements, while TF allows better mapping of the reaction space, allowing complex relationships between variables to be revealed. The response surface areas afforded by the TF model can be used to identify the range of critical process parameters within the design space that can ensure the outcome of the reaction within acceptable limits (QbD), and therefore, they have great potential in the development of safer and more sustainable processes.

## Supporting Information Available

Overview of flow system utilized; detailed description of flow system utilized; description of types of TF experiments performed; and description of models utilized for DoE and transient data (PDF)
