# Supplementary material for: Flash Thermal Racemization of Chiral Amine in Continuous Flow: An Exploration of Reaction Space Using DoE and Multivariate Transient Flow
Source: Org Process Res Dev. 2025 Feb 11;29(2):545–54. doi: 10.1021/acs.oprd.4c00508 (PMC11852205; doi:10.1021/acs.oprd.4c00508)
Supplement: Supplementary file 1 — op4c00508_si_001.pdf [file op4c00508_si_001.pdf]

Flash Thermal Racemization of Chiral Amine in Continuous Flow: An Exploration of Reaction Space using DoE and Multivariate Transient Flow

Matthew J. Takle,[a‡] Linden Schrecker,[a‡§] Benjamin J. Deadman,[b†] Joachim Dickhaut,[c] Andy Wieja,[c] Klaus Hellgardt,[d] King Kuok (Mimi) Hii[a,*]

[a]Department of Chemistry and [b]Centre for Rapid Online Analysis of Reactions, Imperial College London, Molecular Sciences Research Hub, 82, Wood Lane, London, W12 0BZ, UK.

[c]BASF SE, Ludwigshafen 67056, Germany.

[d]Department of Chemical Engineering, Imperial College London, Exhibition Road, South Kensington, London, SW7 2AZ, UK.

Content

S1 Flow system used for transient flow experiments....2
S1.1 System overview....2
S1.2 Pumps....3
S1.3 Tubing and fittings....3
S1.4 Heating and cooling....3
S1.5 System pressure....3
S2 Transient experiments....4
S2.1 Overview....4
S2.2 Temperature Ramp....4
S2.3 Amine Concentration Ramp....5
S2.4 Flow Rate Ramp....5
S2.5 Temperature-Flow Rate Ramp....7
S2.6 Temperature-Amine Concentration Ramp....8
S3 Multivariate polynomial models and data accessibility....9
S3.1 DoE models....9
S3.2 Transient flow models....10
S3.3 Comparison of models & data statement....12
S4 References....12

S1 Flow system used for transient flow experiments

S1.1 System overview

Preparation of the packed bed reactor (PBR): The end of a 1/4" OD SS column (12 cm) was sealed with a 1/4 -inch Swagelok fitting and plugged with glass wool, followed by sand (200 mg). The column was then packed with pre-mixed 5 wt% Pd/Al_2O_3 (200 mg, 60 - 108 micron) and SiC (1 g, 180 micron), before sealing with a layer of sand (200 mg) and glass wool. Residence time was calculated from taking the difference between dry and wet weight of the column, to give a void volume of 1.05 mL (toluene ρ = 0.867 mg/mL). This results in residence times of 63 seconds, 16 seconds and 9 seconds at flow rates of 1 mL min^{-1}, 4 mL min-1 and 7 mL min^{-1} respectively.

Note: 'SS1' tubing, dimensions: 1 mm ID, 1/16" OD; 'SS2' tubing, dimensions, 1.5 mm ID, 1/8" OD.

A solution of the chiral amine in toluene and toluene solvent were placed in round bottom flasks, under nitrogen atmosphere. The flasks are closed using rubber septa. The solutions are delivered via needles pierced through the septa, attached by Luer lock fittings to inlet tubing lines (1/8" OD, 2 mm ID PTFE, 1.6 mL and 1.2 mL respectively) connected to a Gilson 305 HPLC pump (pump A) and a Gilson 307 HPLC pump (pump B). The outlets of these pumps were connected to two lengths of SS tubing (25 cm) which fed into a Valco T-piece stainless steel mixer. The outlet of the mixer is connected to a 25 cm length of SS1 tubing of which the inlet fitting is an HPLC type fitting, and the outlet is Swagelok. This is connected by a 1/16" to 1/8" adaptor to a 40 cm length of SS2 tubing, the last 30 cm is inside the GC oven, connected to the PBR (Fig. S1b). This exits into 12 cm of SS2 tubing, the first 5 cm of which is in the GC oven (the rest of which is unheated). This connects to a further 15 cm of SS2 tubing, which passes through a custom-built cooling system consisting of an aluminium block and a Peltier assembly (Fig. S2, below). This tubing is connected by a 1/8" to 1/16" adaptor to a 20 cm length of SS1 tubing, connected to a 50 cm length of PTFE 1/16" tubing (1 mm ID) fitted with a ¼-28 flat-bottom flangeless ferrules. This is connected to a back pressure regulator which exits into a 90 cm length of PTFE 1/16" tubing. The resultant reaction mixture was collected into 14 mL test tubes using a Pharmacia biopharma Frac-100 Fraction Collector.

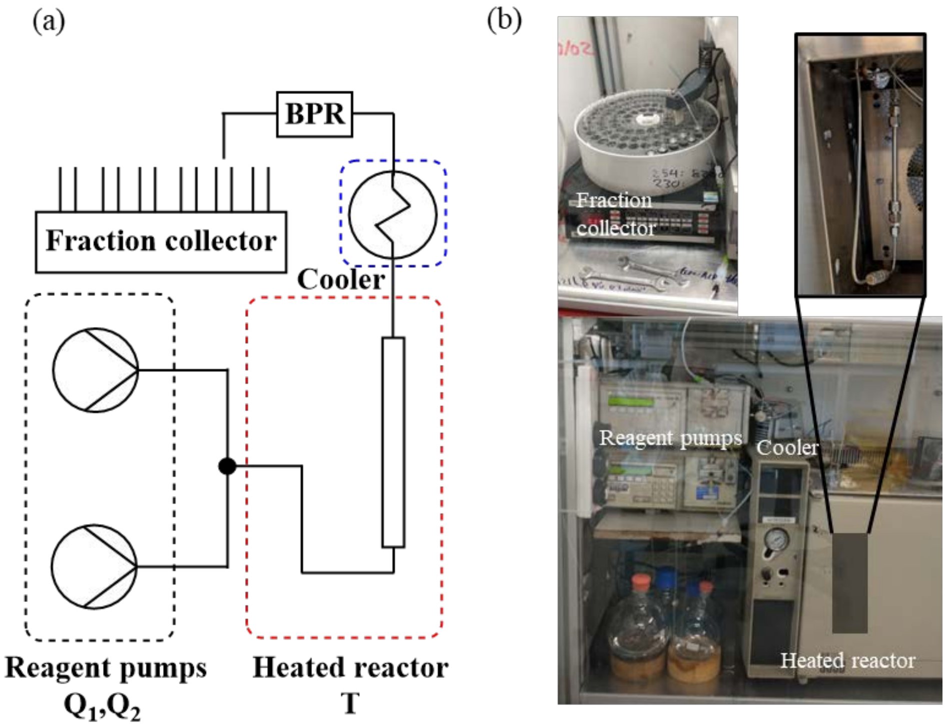

Figure S1. (a) A schematic representation of the flow system constructed for transient flow experiment; (b) Labelled picture of the flow system with inset of the PBR in the GC oven.

S1.2 Pumps

The pumps used are Gilson 305 and 307 HPLC pumps, fitted with a 10 mL WSC and a 10 mL SC pump heads, respectively. These are connected by a GSIOC cable so that the pumps can control each other as a master/slave system through in-built Gilson firmware. In order to maintain the pumps in working order and confirm their accuracy, the check valves are cleaned and sonicated in methanol regularly, and the cumulative flow rate of the system is confirmed before and after experimentation at multiple flow rates.

S1.3 Tubing and fittings

The pump inlets are made of PTFE tubings (1/8” OD, 2 mm ID) with volumes of 1.6 mL and 1.2 mL for pumps A and B respectively. The stainless steel tubing used in the system is either 1/16” (‘SS1’, 1 mm ID, Thames Restek UK Ltd.), or 1/8” stainless steel tubing (‘SS2’, 1.5 mm ID, 1/8” OD). A VALCO T-piece mixer is used to mix the two solutions. The outlet of the back pressure regulator (BPR) is connected to a PTFE tubing (1/16” OD, 1 mm ID). The fittings to the BPR are ¼-28 flat-bottom flangeless ferrules. The inlet and outlet fittings to the Gilson pumps are standard Gilson 30X fittings. All other fittings are procured from Swagelok.

S1.4 Heating and cooling

Heating of the reactor is achieved by repurposing an HP 5890 Series II GC oven, using the in-built software/PID to allow accurate control over temperature ramping. Cooling is performed by a 60 W Peltier thermo-electric cooler module and heatsink assembly (PiHut) with a custom cooling block milled to fit 1/8” tubing by the Advanced HackSpace, Imperial College London (Fig. S2).

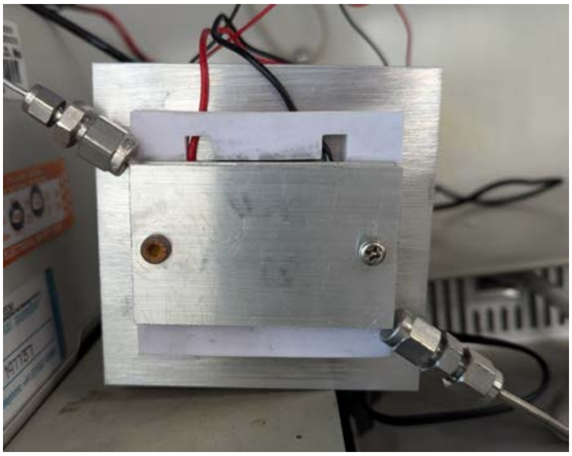

Figure S2. The cooling system used in the flow system consisting of a 60 W Peltier assembly connected to a custom aluminium block containing a cutting which fits to 1/8” stainless steel tubing.

S1.5 System pressure

The back pressure regulator used was an Upchurch Scientific 250 psi BPR. This kept the system at 17 bar; an adequate pressure to maintain toluene in the solution phase across the range of temperatures used.

S2 Transient experiments

S2.1 Overview

The catalyst packed bed was prepared in the same way as for the DoE work.[1] Transient flow experiments were performed across different temperatures (100 – 230 °C), amine concentrations (20.1 – 81.6 mM), and flow rates (1 – 7 mL min^{-1}). All ramps were performed automatically through firmware on the pumps and oven, as detailed under each subsection (below). An initial set of three experiments was performed at the midpoint of two parameters and varying the third parameter as a linear ramp, repeated in the opposite ramping direction. The results of these ramps then informed the selection of two multivariate ramping experiments to perform to gain more insight into the response of selectivity and e.e. within the reaction design space. The autosampler was set to collect aliquots in one-minute intervals. Each collection vial was transferred to a labelled vial, and subsequently diluted using an Opentron automated liquid handler: 20 μL of the aliquot was diluted 50-fold using a solution of anisole (internal standard, 10 mM in methanol). These diluted samples were analysed by GC-FID and chiral HPLC to assess selectivity and e.e., respectively. Analytical methods were previously described.[1]

S2.2 Temperature Ramp

Toluene was used at 4 mL min^{-1} to prime the system as the temperature was increased to 100 °C. A solution of the amine (51.6 mM) was then pumped at 4 mL min^{-1} through the second pump at 100 °C. The method in table S1 was started concurrently with the autosampler.

Table S1. Temperature ramp method utilised.

Experiment time / min	Flow rate / mL min^{-1}	Temperature / °C	Concentration / mM
0	4	100	51.60
15	4	100	51.60
28	4	230	51.60
38	4	230	51.60
51	4	100	51.60
61	4	100	51.60

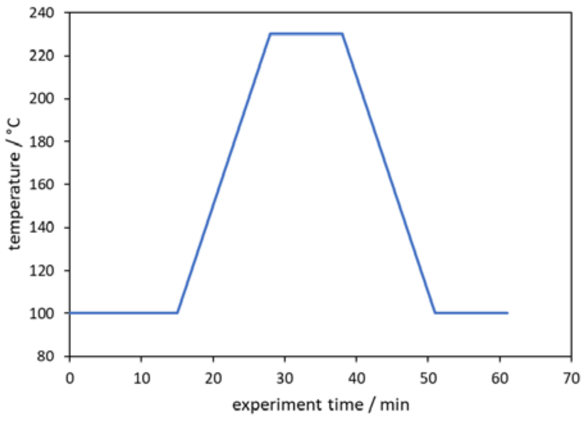

Figure S3. Temperature ramping method.

S2.3 Amine Concentration Ramp

Toluene was used at 4 mL min^{-1} to prime the system through one pump as the temperature was increased to 165 °C. The toluene pump flow rate was reduced to 3 mL min^{-1} and a solution of the amine (81.6 mM) was pumped at 1 mL min^{-1} through the second pump (25%B, 4 mL min^{-1} cumulative flow rate) at 165 °C. The method in table S2 was then started concurrently with the autosampler beginning collecting fractions.

Table S2. Amine concentration ramp method utilised.

Experiment time / min	Flow rate / mL min^{-1}	Temperature / °C	Concentration / mM	%B
0	4	165	20.4	25
10	4	165	20.4	25
23	4	165	81.6	100
33	4	165	81.6	100
46	4	165	20.4	25
56	4	165	20.4	25

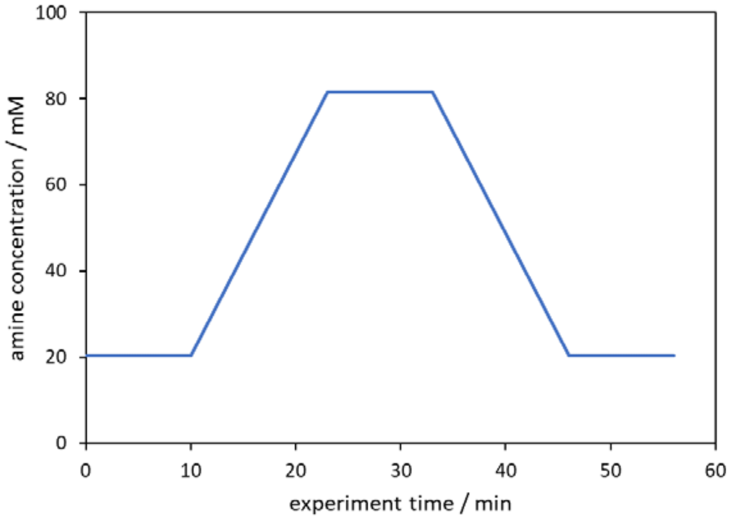

Figure S4. Amine concentration ramping method (inputted to pump method as "%B").

S2.4 Flow Rate Ramp

Toluene was used at 7 mL min^{-1} to prime the system through one pump as the temperature was increased to 165 °C. A solution of the amine (51.6 mM) was then pumped at 7 mL min^{-1} through the second pump at 165 °C and the method in table S3 was started concurrently with the autosampler beginning collecting fractions.

Table S3. Pump flow rate ramp method utilised.

Experiment time / min	Flow rate / mL min^{-1}	Temperature / °C	Concentration / mM
0	7	165	51.6
10	7	165	51.6
23	1	165	51.6
33	1	165	51.6
46	7	165	51.6
51	7	165	51.6

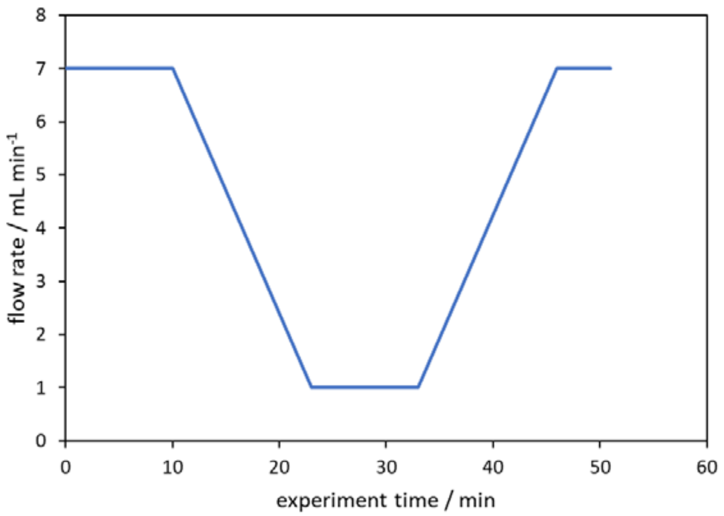

Figure S5. Flow rate ramping method.

Note that only the downwards ramping data was utilised as increasing flow rate ramps are often inaccurate, as discussed in previous work.[2]

S2.5 Temperature-Flow Rate Ramp

Toluene was used at 7 mL min^{-1} to prime the system through one pump as the temperature was increased to 100 °C. A solution of the amine (51.6 mM) was then pumped at 7 mL min^{-1} through the second pump at 100 °C and the method in table S4 was started concurrently with the autosampler beginning collecting fractions.

Table S4. Method utilised for temperature-flow rate multivariate ramps.

Experiment time / min	Flow rate / mL min^{-1}	Temperature / °C	Concentration / mM
0	7	100	51.6
15	7	100	51.6
28	1	230	51.6
37.99	1	230	51.6
38	7	230	51.6
48	7	230	51.6
61	1	100	51.6
71	1	100	51.6

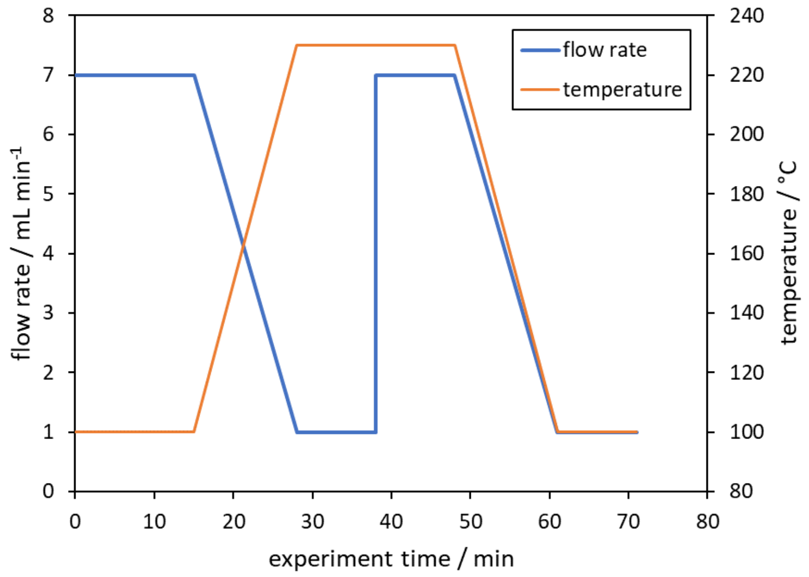

Figure S6. Temperature-flow rate multivariate ramping method.

S2.6 Temperature-Amine Concentration Ramp

Toluene was used at 4 mL min^{-1} to prime the system through one pump as the temperature was increased to 100 °C. The toluene pump flow rate was reduced to 3 mL min^{-1} and a solution of the amine (81.6 mM) was pumped at 1 mL min^{-1} through the second pump (25%B, 4 mL min^{-1} cumulative flow rate) at 100 °C. The method in table S5 was then started concurrently with the autosampler beginning collecting fractions.

Table S5. Method utilised for temperature-concentration multivariate ramps.

Experiment time / min	Flow rate / mL min^{-1}	Temperature / °C	Concentration / mM	%B
0	4	100	20.40	25
15	4	100	20.40	25
28	4	230	81.60	100
33	4	230	81.60	100
43	4	230	20.40	25
48	4	230	20.40	25
61	4	100	81.60	100
71	4	100	81.60	100

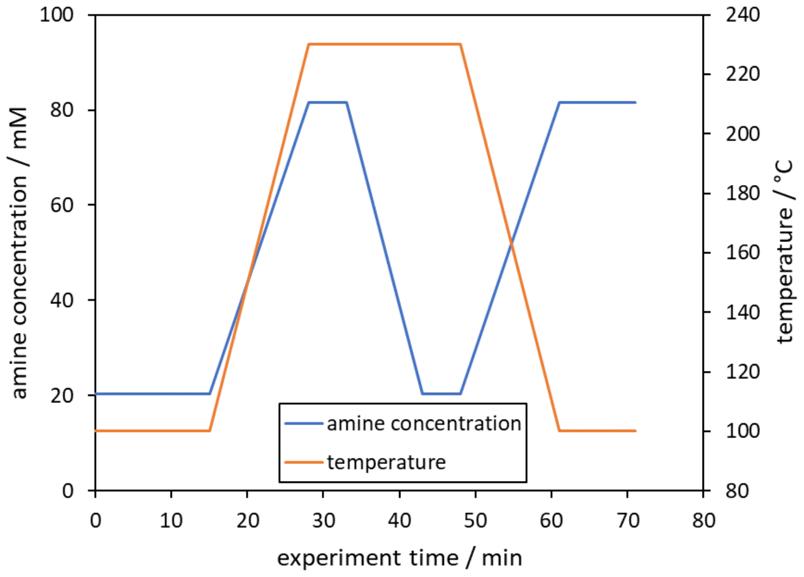

Figure S7. Temperature-amine concentration ramping method (amine concentration method inputted to pump method as “%B”).

S3 Multivariate polynomial models and data accessibility

The data produced in this work is accessible along with the code at https://github.com/LindenSchrecker.

For this section and throughout the code, the following symbols are used: T = temperature; C = concentration; Q = cumulative flow rate.

S3.1 DoE models

The DoE experimental data were utilised in JMP to fit multivariate polynomial fitting with second order terms. The coefficients for each fitted term are outlined for e.e. and selectivity in Table S6. Terms which yield a 0 coefficient were those deemed negligible by the JMP model software. R^2 and RMSE value are for data set that this model was trained on.

Table S6. Coefficients for multivariate polynomial models based on DoE data.

Term	e.e.	Selectivity
(intercept)	448.547	97.18
T	-4.9174	-0.33451
C	0	0.99596
Q	14.2522	3.5667
T^2	0.01317	0
TC	0	-0.00544
TQ	-0.0677	0
C^2	0	0
CQ	0	0
Q^2	0	0
R^2	0.95	0.97
RMSE	8.3313	7.1115

S3.2 Transient flow models

The transient flow experimental data were utilised with a python script (accessible at https://github.com/LindenSchrecker) to fit multivariate polynomial fitting with second order terms. The coefficients for each fitted term are outlined for e.e. and selectivity in Table S7. R^2 and RMSE value are for data set that this model was trained on.

Table S7. Coefficients for 2nd order multivariate polynomial models based on transient flow data.

Term	e.e.	Selectivity
(intercept)	256.449	-86.614
T	-2.109818	1.025507
C	-1.960981	2.387028
Q	0.159679	-0.192159
T^2	0.006223	-0.004568
TC	0.001082	-0.003168
TQ	-0.103031	0.070818
C^2	0.001405	0.001765
CQ	0.395513	-0.367277
Q^2	0.065848	1.561749
R^2	0.9184	0.8512
RMSE	5.281	9.330

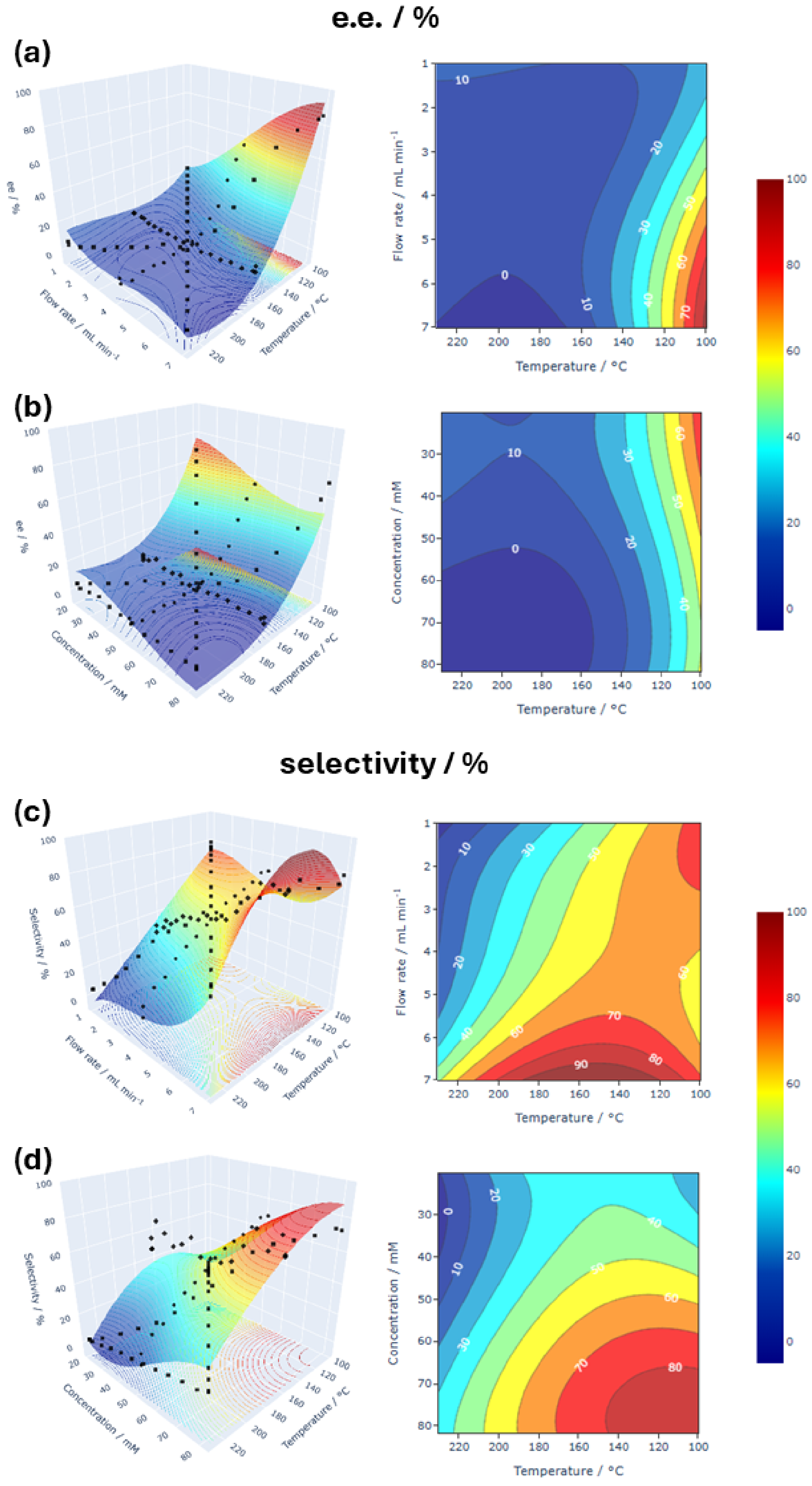

Figure S8. Response Surfaces generated by 3rd order polynomial fitting of transient flow data collected across mono- and bi-variate ramps. Response surfaces (left) and contour plots (right) of the e.e. (a, b) and selectivity (c, d) models (third order).

Table S8. Coefficients for 3rd order multivariate polynomial models based on transient flow data.

Term	e.e.	Selectivity
(intercept)	449.344	-209.848
T	-0.332648	0.179824
C	-0.103690	0.182856
Q	0.000191	-0.000247
T^2	0.017534	-0.004114
TC	-0.042934	-0.024140
TQ	-1.326979	0.721121
C^2	-0.121827	0.158232
CQ	-0.404883	0.718659
Q^2	0.034088	-0.059059
T^3	-0.000031	0.000005
T^2C	-0.000024	0.000059
T^2Q	0.001363	-0.001550
TC^2	-0.000064	0.000103
TCQ	0.014658	-0.002464
TQ^2	0.002442	-0.001691
C^3	0.000299	-0.000475
C^2Q	0.022373	-0.025711
CQ^2	0.099895	-0.121750
Q^3	-0.456373	0.670181
R^2	0.9573	0.8823
RMSE	3.817	8.297

S3.3 Comparison of models & data statement

Comparisons were made between the model based on DoE data and the model based on transient flow data utilising a python script. These comparisons were also visualised in python utilising the plotly package. This produces interactive figures which can be accessed through the script at https://github.com/LindenSchrecker.

The reaction data will be added to the Open Reaction Database (https://open-reaction-database.org and https://github.com/open-reaction-database/ord-data) with the dataset ids of ord_dataset-d9140e7c806047b78bcbbc85cfd5b7fc for the DOE data, and ord_dataset-3d64cf72f75d4b998411f352a1d3f909 for the TF data.

S4 References

1 "Flash Thermal Racemization Protocol for the Chemoenzymatic Dynamic Kinetic Resolution and Stereoinversion of Chiral Amines", M. J. Takle, B. J. Deadman, K. Hellgardt, J. Dickhaut, A. Wieja and K. K. M. Hii, *ACS Catal.*, 2023, **13**, 10541–10546.

2 "A comparative study of transient flow rate steps and ramps for the efficient collection of kinetic data", L. Schrecker, J. Dickhaut, C. Holtze, P. Staehle, M. Vranceanu, A. Wieja, K. Hellgardt and K. K. Hii, *React. Chem. Eng.*, 2024, **9**, 1077–1086.
